# Shaping the Future of Older Adult Care: ChatGPT, Advanced AI, and the Transformation of Clinical Practice

**DOI:** 10.2196/51776

**Published:** 2023-09-13

**Authors:** Kathleen Fear, Conrad Gleber

**Affiliations:** 1 UR Health Lab, University of Rochester Medical Center Rochester, NY United States; 2 Department of Medicine, University of Rochester Medical Center Rochester, NY United States

**Keywords:** generative AI, artificial intelligence, large language models, ChatGPT, Generative Pre-trained Transformer

## Abstract

As the older adult population in the United States grows, new approaches to managing and streamlining clinical work are needed to accommodate their increased demand for health care. Deep learning and generative artificial intelligence (AI) have the potential to transform how care is delivered and how clinicians practice in geriatrics. In this editorial, we explore the opportunities and limitations of these technologies.

## Introduction

The older adult population in the United State is ballooning: by 2030, one in 5 Americans will be aged 65 years or older, and by 2060, that number will climb to nearly 1 in 4 [1]. As this demographic grows, their need for health care will increase as well. At the same time, 1 in 5 doctors and 2 in 5 nurses say they are likely to leave clinical practice in the next 5 years, whereas 1 in 3 physicians, advance practice providers, and nurses intend to reduce their current working hours [2]. The World Health Organization projects a shortfall of up to 10 million health care workers globally by 2030 [3]. To close this impending gap between health care needs, especially in older adult care, and available clinical resources, it is imperative that health care be fundamentally reimagined.

## Opportunities in Generative AI

Deep learning, and large language models (LLMs) in particular, offer promise in their potential to transform how clinicians work to meet the health care needs of the older adult population. LLM applications such as ChatGPT (OpenAI) [4] have a unique ability to create humanlike responses from a conversational prompt, opening new possibilities for interacting with and generating insights from data, streamlining everyday tasks, and automating routine work for clinicians. Early work has explored the effectiveness of LLMs in facilitating activities that are burdensome and time-consuming but require relatively little actual clinical decision-making, such as managing messages and work tasks in the communication hub of the electronic health record (EHR) system [5]. Researchers at the University of California, San Diego demonstrated that ChatGPT could effectively respond to patient messages: a group of health care professionals was asked to review ChatGPT’s responses to questions along with physicians’ responses to the same questions, and they consistently rated ChatGPT’s responses as higher quality and more empathetic than those composed by the physicians [6]. Several other institutions are trialing the same approach, independently or in partnership with EHR providers [7].

Some of the most exciting applications of generative AI might be those that use these tools to boost clinical reasoning and decision-making. LLMs can take in and synthesize immense amounts of unstructured data. This means that nearly everything in EHRs could be used by LLMs in an analysis, including clinical notes, lab results, imaging scans, genetic information, and patient-generated health data. For example, it can be challenging for a busy hospitalist to distill a patient's entire chart during admission. Yet through experience, clinicians learn to prioritize which part of a patient’s story has the highest yield. Combining this clinical expertise with LLM-based tools can help identify patterns, correlations, and subtle relationships in the clinical data that may not be immediately apparent. As a result, this approach can help clinicians to work more efficiently and effectively and make more accurate and data-driven diagnoses. LLMs can also help identify patterns associated with high-risk patients with chronic conditions to facilitate the development of personalized preventive care strategies [8].

Another the top innovation priority in health care is the patient experience. ChatGPT can provide valuable information and support to older adults who often face health challenges and need assistance in personal and health care [9]. Generative AI–powered chatbots and virtual assistants can help remotely monitor high-risk older adults with multiple chronic conditions and provide personalized health, nutrition, and fitness advice to help them manage their conditions [10]. Through a sense of virtual companionship, connections, and nonjudgmental emotional support, ChatGPT can also help address social isolation and loneliness in older adults [11,12]. Creative applications of generative AI to advance health care for older adults, including remote health monitoring, mental health support, and personalized prevention of cognitive decline, have been increasingly explored in the literature and are expected to demonstrate effects and impacts in the future [13-16].

## Potential Risks and Limitations

To err is human. Likewise, despite the incredible abilities of these technologies, no predictive or generative model will perform perfectly. It is critical to understand the sources of bias and errors in AI tools and develop realistic benchmarks for safe performance. For instance, the training data for the largest current LLMs are mostly “general knowledge”: these models are trained using a huge and broad data set sourced from the internet. As a result, these models excel at a wide variety of tasks, but they can fall short when specialized medical knowledge is required [17,18]. Disconcertingly, these models can fail in ways that are misleading or nonobvious, which raises concerns regarding the ability of these models to support clinical decision-making [19]. Further, using these models can come with a substantial cost, including either the direct cost to access them via a vendor or third-party platform or the development, implementation, or maintenance costs for internally building open-source products [20]. Finally, many LLMs are energy and resource intensive to run, raising substantial concerns about the environmental impact of a large-scale adoption of these tools [21].

## Conclusion

Deep learning and generative AI have the potential to transform health care; if used well, as they are incorporated into clinical workflows, they could fundamentally change how clinicians practice. As the population ages and demand for care increases, the sustainability of health care depends on developing new, smarter, and more effective ways of managing the routine and complex tasks that make up clinicians’ day-to-day work while facilitating high-quality care and support for the older adult population.

## Abbreviations

AI: artificial intelligence
EHR: electronic health record
LLM: large language model
